# Sepsis-Induced Takotsubo Cardiomyopathy Leading to Torsades de Pointes

**DOI:** 10.1155/2016/2384752

**Published:** 2016-07-20

**Authors:** Nirav Patel, Abhishek Shenoy, George Dous, Haroon Kamran, Nabil El-Sherif

**Affiliations:** ^1^Department of Medicine, SUNY Downstate Medical Center, Brooklyn, NY 11203, USA; ^2^Department of Cardiology, VA NY Harbor Healthcare System, Brooklyn, NY 11209, USA; ^3^College of Medicine, SUNY Downstate Medical Center, Brooklyn, NY 11203, USA; ^4^Department of Cardiology, SUNY Downstate Medical Center, Brooklyn, NY 11203, USA

## Abstract

*Background*. Takotsubo cardiomyopathy (TCM) is sudden and reversible myocardial dysfunction often attributable to physical or emotional triggers.* Case Report*. We describe a 51-year-old man presented to emergency department with sepsis from urinary tract infection (UTI). He was placed on cefepime for UTI and non-ST-elevation myocardial infarction protocol given elevated troponins with chest pain. Subsequently, patient was pulseless with torsades de pointes (TdP) and then converted to sinus rhythm with cardioversion. An echocardiogram revealed low ejection fraction with hypokinesis of the apical wall. Over 48 hours, the patient was extubated and stable on 3 L/min nasal cannula. He underwent a cardiac catheterization to evaluate coronary artery disease (CAD) and was found to have mild nonobstructive CAD with no further findings.* Conclusion*. TCM is a rare disorder presenting with symptoms similar to acute coronary syndrome. Though traditionally elicited by physical and emotional triggers leading to transient left ventricular dysfunction, our case suggests that it may also be triggered by a urinary tract infection and lead to severe QT prolongation and a malignant ventricular arrhythmia in TdP.

## 1. Introduction

Takotsubo cardiomyopathy (TCM) is an abrupt and often unpredictable presentation of an acute heart failure syndrome with symptoms of chest pain and shortness of breath, often triggered by an emotionally or physically stressful event. The disease is characterized by transient systolic and diastolic left ventricular dysfunction with a diverse range of described wall motion abnormalities [1]. TCM, also known as broken-heart syndrome, is most often seen in elderly women, with Asians being the largest group affected. It presents with some sort of emotional or physical trigger leading to symptoms of acute coronary syndrome including chest pain, shortness of breath accompanied by echocardiographic findings, and biomarker profiles suggesting TCM. Although it has been observed worldwide over the past 26 years since its original description in Japan, there is still much that is unknown about this acute illness [2]. We would like to present a case of sepsis-induced TCM leading to QTc prolongation which in turn led to a malignant ventricular arrhythmia in torsades de pointes (TdP). In the literature search, there are cases presented with TCM and TdP; however, there is no case report mentioning of sepsis-induced TCM leading to TdP.

## 2. Case Report

We present a case of a 51-year-old white man who initially presented with substernal chest pain to the emergency department and was found to be in sepsis. Upon presentation, the patient was found to be tachycardic and hypotensive, accompanied by a leukocytosis. He was diagnosed with sepsis secondary to a urinary tract infection (UTI). Urine cultures were sent but empiric treatment was begun with cefepime based on his previous history of recurrent urinary tract infection with* Pseudomonas aeruginosa* sensitive to cefepime.

**Figure 1:**
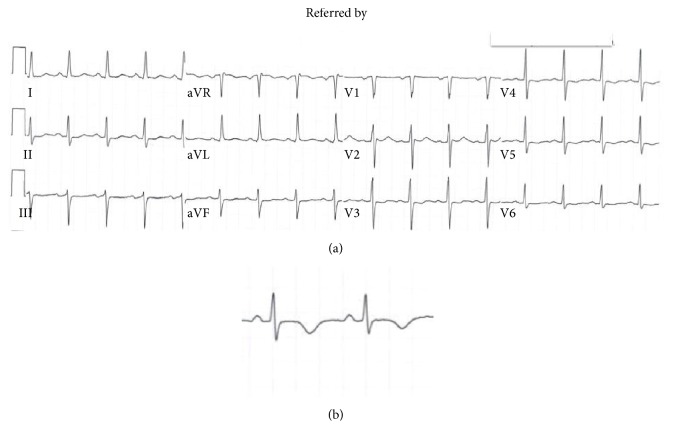
(a) EKG on admission with normal QTc of 446 ms. (b) Close-up of lead V2.

**Figure 2:**
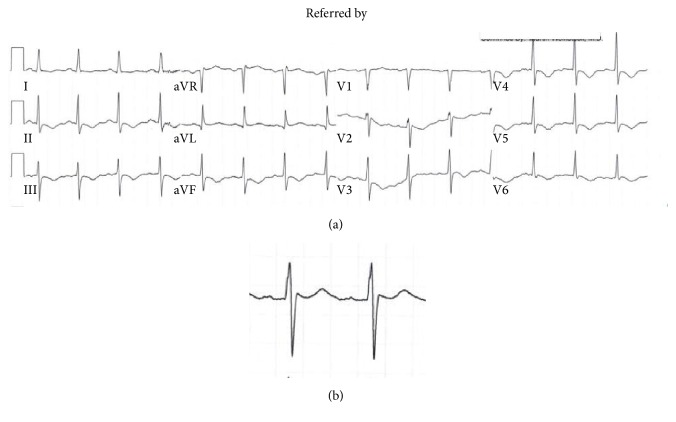
(a) After 12 hours of admission, progressive T wave inversion; the QTc is being prolonged of 491 ms. (b) Close-up of lead II.

On admission, cardiac biomarkers were also sent for analysis and troponin ultra was found to be elevated to 3.312 ng/mL (reference range 0.006–0.06 ng/mL), without any ST-T wave changes. On admission, leukocyte counts were elevated 15,800/*μ*L and also lactate was 2.1 mmol/L. The patient was subsequently admitted to the coronary care unit and placed on aspirin, clopidogrel, and enoxaparin, treating him for a possible non-ST elevation myocardial infarction. The patient was not started on a beta-blocker due to hypotension. Since admission, the patient had progressive T wave changes (Figures 1 and 2).

**Figure 3:**
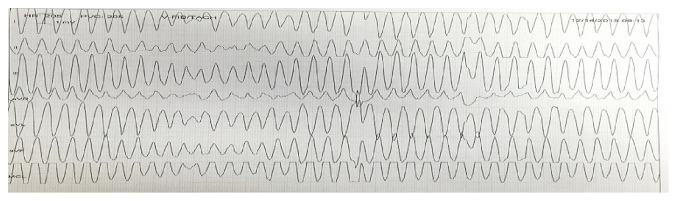
After 22 hours of admission, telemetry strip showing TdP.

**Figure 4:**
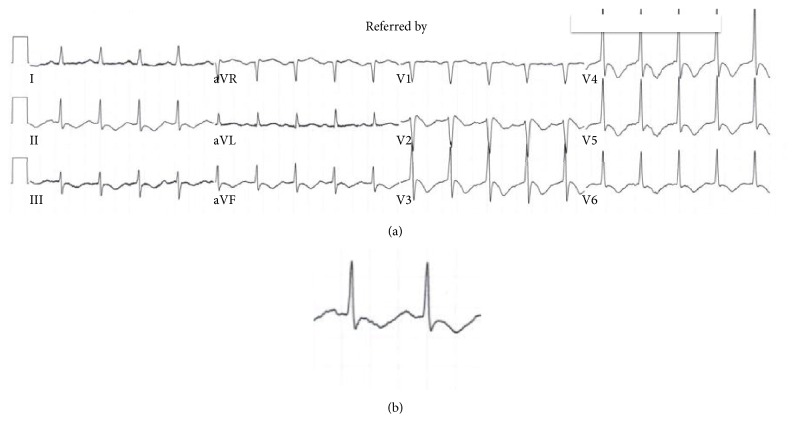
(a) After TdP, progressive T wave inversion and QTc prolongation of 691 ms. (b) Close-up of lead II.

For the purpose of this case, the patient's past medical history consisted of a neurogenic bladder with recurrent UTIs, recurrent deep vein thrombosis on coumadin, multiple sclerosis, hypertension, and hyperlipidemia. After the patient was admitted to the coronary care unit, the patient was found to be in torsades de pointes (TdP) pulseless polymorphic ventricular tachycardia (Figure 3). The patient was successfully electrically cardioverted. After cardioversion, T wave inversions worsened with evidence of prolonged QTc (Figure 4). The patient was intubated to maintain and protect airways.

Subsequently, the patient was started on pressor support to maintain his mean arterial pressure greater than 65 mmHg. The following day, his mental status improved and he was tolerating a continuous positive airway pressure trial. He was then extubated and found to be stable on 3 L nasal cannula.

**Figure 5:**
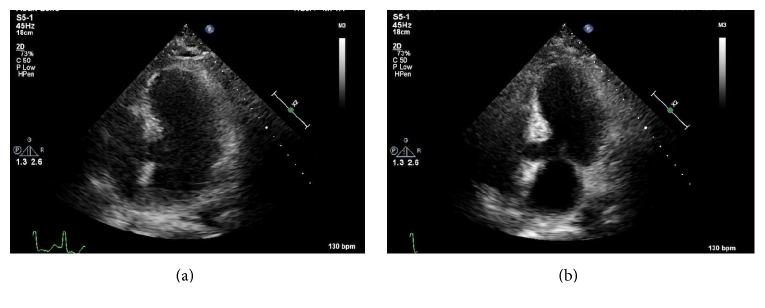
Echocardiographic evidence of classic takotsubo cardiomyopathy, the apical type. (a) represents diastole and (b) represents systole.

**Figure 6:**
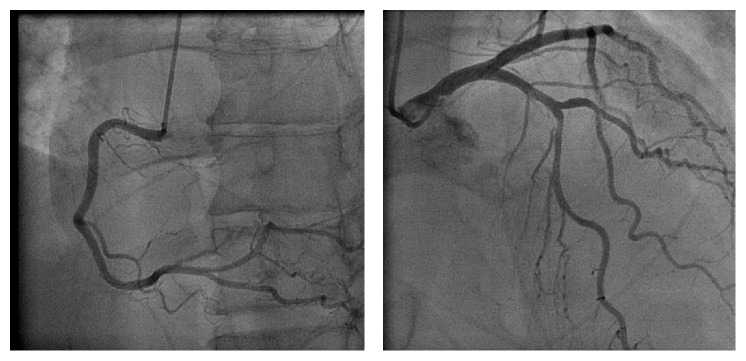
Angiogram reveals normal coronary arteries.

After the patient was hemodynamically stable, the plan was to gather more information on the underlying nature behind his TdP and long QT. The patient was scheduled for a transthoracic echocardiogram (TTE) and a cardiac catheterization the following day. The TTE revealed a reduced left ventricular ejection fraction (25%–30%) with apical wall motion abnormalities consistent with a great likelihood of classic takotsubo cardiomyopathy, the apical type (Figure 5). A coronary angiogram was performed a few days later and did not show any significant coronary artery disease (Figure 6).

**Figure 7:**
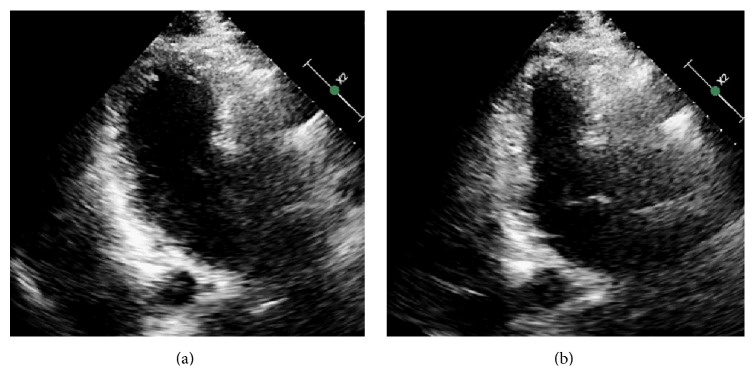
Echocardiographic evidence of normalization of left ventricular ejection fraction. (a) represents diastole and (b) represents systole.

This case shows TCM in the setting of sepsis secondary to UTI. Urine culture was positive for* Pseudomonas putida and Escherichia coli* on day 3 of admission and blood cultures were negative. We believe that the TCM in this patient led to a malignant ventricular rhythm in TdP, which eventually needed direct current cardioversion to resolve. The patient's hospital course was stable following the treatment, with a resolving white cell count and a treated UTI. The patient was discharged with a life vest. Patient had repeated echocardiogram three months later and left ventricular ejection fraction improved to >55% without any wall motion abnormality (Figure 7).

## 3. Discussion

Takotsubo cardiomyopathy (TCM), also known as broken-heart syndrome, was first described more than 25 years ago; however, it has still not been completely understood. TCM is usually an acute, distinct cardiomyopathy with transient left ventricular dysfunction and mimics myocardial infarction without significant coronary obstruction [1, 3, 4]. However, TCM may have relevant coronary artery obstruction but the extent of myocardial dysfunction far exceeds the culprit stenotic lesion or it can be different stenotic lesion compared to the myocardial dysfunction [1, 5]. Its symptomology (chest pain, palpitations, and shortness of breath) overlaps with that of acute coronary syndrome and therefore is a serious medical event [4]. Original reports on TCM done in Japan, the United States, and Western Europe describe individuals with transient left ventricular dysfunction but the condition was not described as a separate condition until Japanese clinicians did so in the 1990s [1].

Based on EKG findings, elevated cardiac enzymes, and ACS symptoms, a clinical and lab-based diagnosis of TCM is difficult, because ACS cannot be ruled out. It is usually a reversible event and serial echocardiogram assessments show improvement in left ventricular function. Physical and emotional disturbances trigger TCM 36% and 27.7% of the time, respectively, although, in 28.5% of cases, an identifiable trigger can be absent [1]. The condition usually follows a physical or emotional stressor thought to be a reaction to catecholamine-mediated myocardial stunning [1, 6]. In addition, physical stress is more common in male patients [6]; in this patient, sepsis can be considered a physical trigger.

Although TCM is thought to be a benign and reversible condition, it has been associated with significant morbidity and mortality even though the majority cases of TCM show no angiographic evidence of significant obstructive coronary artery disease [7]. In comparison with acute coronary syndrome, in-hospital complications were similar, about 21.8% [1].

It is the repolarization abnormalities associated with TCM that have been linked to arrhythmias and associated with atrial and ventricular arrhythmias [6, 8]. Atrial fibrillation was the most common arrhythmia, reported in 4.7% of cases, with sinus node dysfunction (in 1.3% cases), atrioventricular nodal dysfunction (including complete heart block in 2.9% cases), and life threatening ventricular tachycardia and fibrillation in 3.4% cases [6]. The direct effect of catecholamines, dysregulation of calcium homeostasis, and oxidative stress are thought to be the contributing factors for the ventricular arrhythmia [6].

TCM has a clinical profile similar to acute coronary syndrome especially in an electrical sense, often initially presenting with ST changes and in the following 24–48 hours presenting with T wave inversions and QT prolongation [6, 9]. For this reason, studies have shown that TCM should be considered a cause of acquired long QT syndrome [6, 9]. Brown et al. [6] described a study showing that there was 8.6% prevalence of life threatening ventricular arrhythmias in patients presenting with TCM. The TCM patients with adverse ventricular arrhythmias as a result presented with a longer QTc on admission and their QT prolongation was more severe as their hospital course progressed [9, 10].

In patients with TCM, there are many risk factors, including genetic, which predispose the person to a prolonged QT interval. For example, a genetic predisposition is present in those with congenital long QT syndrome. This is a syndrome that undoubtedly predisposes a patient to a malignant ventricular rhythm, regardless of whether they have TCM or not, to develop TdP. In addition, electrolyte abnormalities, including hypokalemia (defined as <3.5 mmol/L) and severe hypomagnesemia (defined as <1 mg/dL), have been associated greatly with TCM, and recent conversion of atrial fibrillation to a sinus rhythm [11] and administering QT prolonging agents have all been well-studied risk factors for TdP among TCM patients. These electrolyte abnormalities were present in patients with TCM and were therefore a great contributor to the development of TdP; however our patient's comprehensive metabolic panel was unremarkable for significant electrolyte abnormalities. In addition, cefepime and other cephalosporins have not been shown to prolong QT and are a much safer option than antimicrobials from the macrolides and quinolones families [12].

Though TCM has been linked to QT prolongation and torsades de pointes (TdP) [6, 9], there have not been any clear case reports linking a sepsis-induced TCM that lead to QT prolongation and TdP.

Our patient presented to the emergency department with substernal chest pain and was found to be in sepsis. Within 24 hours of admission, the patient was in TdP. Initially, we could not determine the cause of the steep decline in the patient's cardiac status and it was believed to be ischemic cardiomyopathy. However, transthoracic echocardiography revealed classic apical TCM and gave us a source for the prolonged QT and TdP. The patient's urinary tract infection was the source of his systemic bacterial infection and sepsis, which, we believe, led to a stress-induced cardiomyopathy and TdP. At hospitals without cardiac catheterization laboratories, a high index of suspicion is required to diagnose TCM [13].

The patient was diagnosed with TCM on the basis of the Mayo Clinic diagnostic criteria, of which 3 out of the 4 required diagnostic criteria were met with tested evidence and the fourth criterion was met through clinical judgment. The patient had (1) transient left ventricular systolic dysfunction with a markedly reduced ejection fraction, with hypokinesia and wall motion abnormalities; (2) an absence of acute obstructive coronary disease as cardiac catheterization which showed no evidence of acute plaque rupture or acute coronary syndrome; (3) new electrocardiographic abnormalities including T wave inversions and a prolonged QT interval with a modest elevation in cardiac troponin. The last diagnostic criterion (4) includes excluding pheochromocytoma and myocarditis as an underlying cause of the patient's presentation. Clinically, both these diagnoses were excluded. As the patient did not present with episodic hypertension, palpitations, and excessive diaphoresis, pheochromocytoma was clinically ruled out [14]. As the patient's history did not consist of a recent viral illness, we had a lower suspicion for viral myocarditis. We understand that urine metanephrines, CT abdomen and pelvis, and viral titers could have been ordered to definitively rule out these two conditions before making the diagnosis of TCM; however, our clinical suspicion was low and the acute treatment of our patient was of greater concern. It is important to note that coronary artery disease can be considered nonsignificant in the absence of ≥50% diameter stenoses by visual assessment and angiography [7]. As a result, it is true that nonsignificant coronary artery disease may be present in the presence of TCM; however, this is a finding that is less common (15%) compared to the 85% of TCM patients having normal coronary arteries [7].

## 4. Conclusion

This unique presentation of our patient, initially presenting with sepsis, later progressing to takotsubo cardiomyopathy, subsequently leading to cardioelectrophysiological decompensation and torsades de pointes, illustrated the presence of this very important cause of an acute heart failure syndrome. Moreover, not only can takotsubo cardiomyopathy present with symptoms similar to acute coronary syndrome, but also a clinical presentation in a man without a physical or emotional trigger cannot rule out the complexity of this disease process. Although the pathogenesis of this condition is still not widely accepted, it is important to bear in mind TCM's resemblance to acute coronary syndrome and the potentially lethal complications of this clinical syndrome.
